# From acute immune storm to lifelong vascular risk: a paradigm shift in Kawasaki disease as a chronic disease model

**DOI:** 10.3389/fped.2026.1764216

**Published:** 2026-03-30

**Authors:** Xiong-xiong Yi, Wen-rong Zhang, Dong-mei Wang, Xiu-ping Wang, Fen-xia Zhang

**Affiliations:** 1 Department of Pediatrics, Yan’an People’s Hospital, Yan’an, China; 2 Department of Neonatology, Yan’an People’s Hospital, Yan’an, China

**Keywords:** atherosclerosis acceleration, cardiovascular risk, chronic disease model, endothelial dysfunction, immune dysregulation, Kawasaki disease

## Abstract

Based on accumulating evidence, this article proposes redefining Kawasaki disease as a chronic condition that begins with an acute immune response and leads to lasting vascular and immune dysfunction. This perspective moves beyond the traditional view of Kawasaki disease as a “self-limited vasculitis,” emphasizing that its pathological processes continue long after acute symptoms resolve. The disease follows a continuum involving persistent immune activation, ongoing low-grade inflammation, impaired blood vessel function, and metabolic changes. This chronic disease model explains why patients face accelerated atherosclerosis and higher long-term cardiovascular risk. The article systematically reviews the biology of the disease from its acute to chronic stages, summarizes supporting clinical and imaging evidence, and discusses the implications for lifelong patient management, personalized treatment strategies, and future research across multiple disciplines.

## 1 Introduction: a cognitive paradigm in urgent need of shift

### 1.1 Limitations of the traditional paradigm

The dominant paradigm in clinical practice and research has long defined Kawasaki disease as an “acute, self-limited vasculitis, with coronary artery aneurysms as its primary complication” (1). This framework has successfully guided acute-phase anti-inflammatory therapy and reduced early giant coronary aneurysm formation (2). However, long-term follow-up studies reveal significant limitations. The model fails to adequately explain key observations: even without coronary aneurysms, Kawasaki disease survivors exhibit a higher lifelong risk of cardiovascular events (3). Evidence also indicates persistent immune dysregulation and chronic subclinical endothelial dysfunction beyond the acute phase (4, 5). Thus, viewing the disease solely as a self-limited acute event represents a fundamental conceptual constraint.

### 1.2 Basis for a new paradigm

Emerging evidence supports reconceptualizing Kawasaki disease as a chronic condition. A coherent pathophysiological continuum links the initial systemic hyperinflammation to lasting immune alterations (6). After clinical recovery, a state of low-grade immune activation and chronic inflammation often persists (6). This leads to sustained vascular endothelial dysfunction, which impairs vasoregulation and promotes a pro-atherogenic environment (7). Collectively, these processes accelerate atherosclerosis and increase long-term cardiovascular risk, supporting a chronic disease model (8).

### 1.3 Perspective of this article

**Figure 1:**
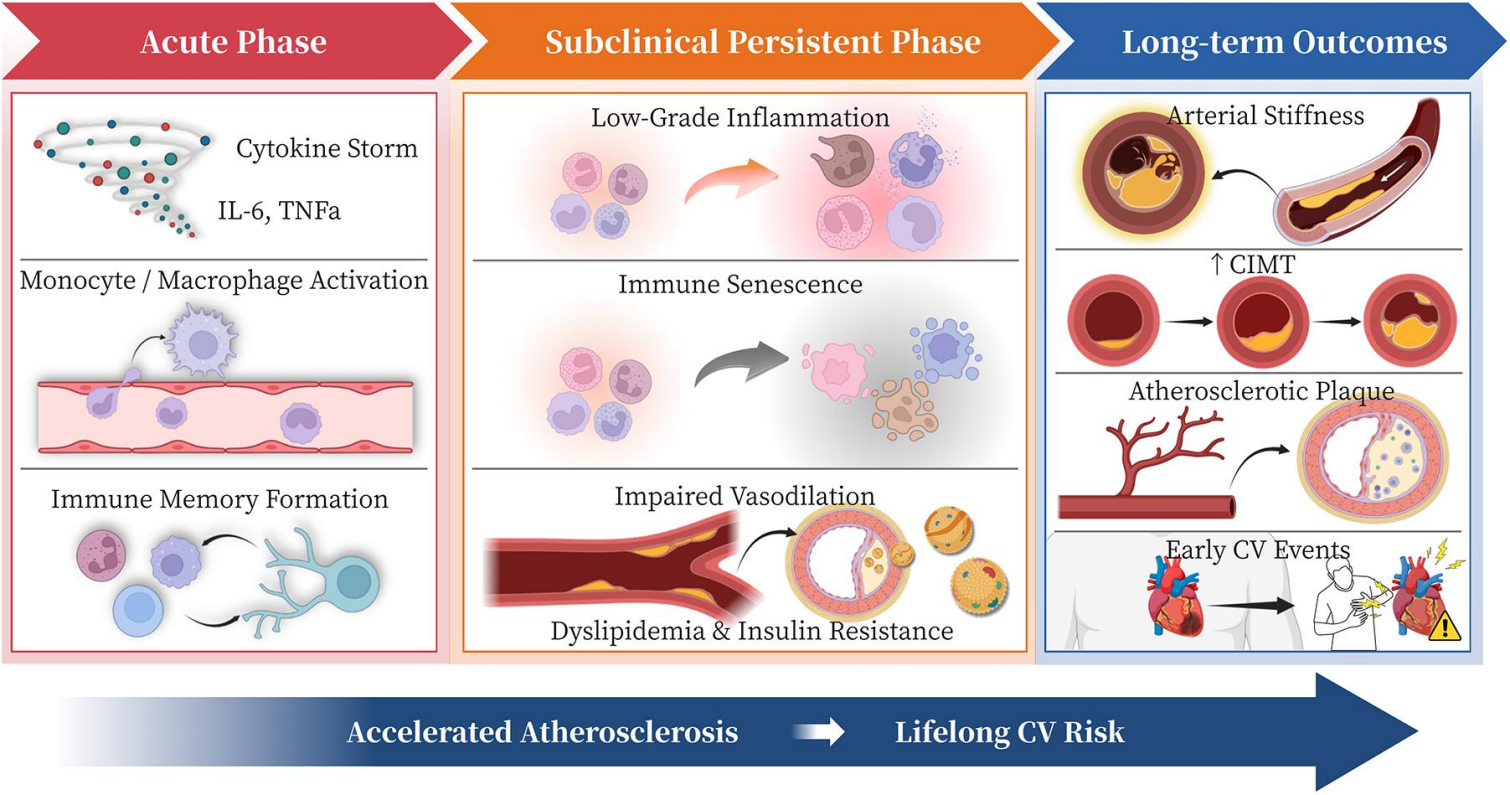
From acute nimmune storm to lifelong cardiovascular risk: a chronic continuum of kawasaki disease. IL-6, interleukin-6; TNFα, tumor necrosis factor-alpha; CIMT, carotid intima-media thickness; CV, cardiovascular; KD, Kawasaki disease. This figure depicts how acute hyperinflammation triggers persistent immune and endothelial dysfunction, leading to subclinical vascular injury, accelerated atherosclerosis, and premature cardiovascular events. This continuum reframes Kawasaki disease as a chronic immune-vascular disorder requiring lifelong surveillance and preventive management.

This perspective article aims to systematically develop the “Kawasaki disease as a chronic disease” paradigm. We first integrate current evidence on its pathophysiology, from immune activation to vascular injury. Next, we review clinical and epidemiological data on long-term cardiovascular outcomes. Finally, we discuss how this paradigm shift transforms clinical management toward lifelong cardiovascular monitoring and tailored interventions. This reframing also opens new research directions in risk stratification and chronic-phase therapeutics, ultimately improving lifelong patient care (Figure 1).

### 1.4 Literature identification and evidence basis

This perspective article is grounded in a structured, non-systematic synthesis of the current literature. We searched major biomedical databases, including PubMed and Web of Science, with emphasis on clinical guidelines, longitudinal cohort studies, and research in areas such as mechanistic immunology, vascular biology, and advanced cardiovascular imaging relevant to both the acute and chronic phases of Kawasaki disease. Priority was placed on high-quality studies that provide insight into long-term outcomes, endothelial function, immune persistence, and cardiovascular risk trajectories. Rather than pursuing exhaustive inclusion, we sought to integrate representative and conceptually influential studies that collectively support reframing Kawasaki disease from an acute vasculitis model toward a chronic disease framework. This approach aligns with the purpose of a perspective article, which is to reinterpret existing evidence, identify emerging patterns, and propose directions for future translational and clinical research.

## 2 Pathophysiological basis: From acute storm to chronic aftermath

### 2.1 Core mechanisms of the acute “immune storm”

The acute phase of Kawasaki disease involves a severe systemic inflammatory response. This begins with excessive activation of the innate immune system, particularly monocytes and macrophages, which triggers a cytokine storm characterized by elevated levels of interleukin-1, interleukin-6, and tumor necrosis factor-alpha. This leads to a state of systemic hyperinflammation (9, 10). The adaptive immune system also becomes actively involved, as dendritic cells present unknown antigens and drive the proliferation and differentiation of antigen-specific T cells and B cells (11, 12). This process establishes long-term immune memory. Importantly, vascular endothelial cells are not passive targets; they become activated by inflammatory mediators, upregulate adhesion molecules, promote leukocyte infiltration, and enter a pro-inflammatory, pro-thrombotic state (13). This acute injury initiates a cascade of chronic pathological changes.

### 2.2 Persistent dysregulation in the “immune-vascular-metabolic” axis during the subclinical phase

After clinical symptoms resolve, a state of persistent immune, vascular, and metabolic dysregulation often continues. Immunologically, patients may show signs of early immune aging, such as reduced *T*-cell diversity. Chronic low-grade inflammation persists, indicated by sustained elevation of cytokines like interleukin-1β and interleukin-6 (6). Recent high-resolution immunological investigations further indicate that the inflammatory imprint established during the acute episode may endure at the cellular scale (14, 15). These studies describe long-lasting reprogramming of circulating monocytes together with persistent perturbation of endothelial–immune communication networks, providing a mechanistic substrate for continued vascular vulnerability (9, 16). Memory immune cells formed during the acute phase can also remain and migrate to the vascular wall, potentially maintaining a low-level inflammatory state (6). Vascular dysfunction becomes a central issue, featuring impaired blood flow-mediated vasodilation and reduced function of endothelial repair cells, which hinders recovery of normal vascular function (6, 17). Metabolically, changes such as altered lipoprotein composition and reduced insulin sensitivity may occur, creating an environment that favors the development of atherosclerosis.

### 2.3 Long-term phase: accelerated vascular aging and atherosclerosis

These sustained changes collectively promote accelerated vascular aging and early atherosclerosis. The process progresses from chronic endothelial dysfunction to structural changes in blood vessels, including increased arterial stiffness (18, 19). Epidemiological and imaging studies confirm that Kawasaki disease survivors have a higher risk and earlier onset of atherosclerosis—such as carotid artery thickening and plaque formation—compared to the general population (7, 19). A key distinction from typical atherosclerosis is the stronger role of inflammation in driving this process in Kawasaki disease patients (3). The underlying pathology is fundamentally linked to the initial immune activation and the lasting vascular dysfunction it causes. Recent translational evidence indicates that vascular injury in Kawasaki disease represents a chronic remodeling process rather than a fully reversible event (9, 17, 20). This evolving understanding underscores the need for long-term cardiovascular surveillance.

## 3 Clinical and translational evidence supporting the new paradigm

### 3.1 Insights from longitudinal cohort studies

Long-term follow-up studies provide critical population-level evidence for the chronic disease model of Kawasaki disease. Multiple large-scale longitudinal cohorts indicate that even individuals who recovered from Kawasaki disease without coronary artery aneurysms exhibit significantly poorer long-term cardiovascular health compared to their peers (1). Specifically, these individuals demonstrate a higher incidence of atherosclerotic cardiovascular events in adulthood (21). Additionally, the prevalence of hypertension, dyslipidemia, and metabolic syndrome is markedly increased, with an earlier age of onset (22). These findings strongly suggest that Kawasaki disease induces systemic and persistent pathological changes beyond acute coronary injury.

### 3.2 The evolving trajectory of biomarkers

Biomarker dynamics illustrate the disease's progression from acute to chronic phases. During acute illness, traditional inflammatory markers such as C-reactive protein are significantly elevated (23). In the chronic phase, low-grade inflammation persists, evidenced by markers like high-sensitivity C-reactive protein (23). Concurrently, indicators of vascular function and remodeling show lasting alterations. For example, adiponectin levels may remain low, while matrix metalloproteinase activity shows prolonged dysregulation (24, 25). This biomarker trajectory provides objective evidence for the disease's chronic progression.

### 3.3 Advanced imaging evidence

Modern non-invasive imaging reveals subclinical vascular abnormalities in Kawasaki disease survivors. Coronary computed tomography angiography can detect coronary wall thickening and non-obstructive plaques (26). Increased carotid intima-media thickness, measured via ultrasound, indicates early systemic atherosclerosis (27). Elevated arterial stiffness, assessed by pulse wave velocity, reflects impaired vascular function (28). These imaging findings depict accelerated vascular aging, often preceding clinical symptoms.

### 3.4 Analogies from other chronic inflammatory disease models

The chronic vascular pathology of Kawasaki disease shares mechanisms with other systemic inflammatory diseases. Patients with rheumatoid arthritis or systemic lupus erythematosus also experience accelerated atherosclerosis exceeding traditional risk predictions (29). These conditions share a common pathway: chronic immune inflammation leading to endothelial dysfunction and atherosclerosis (30). In all, persistent inflammation damages the endothelium and promotes plaque formation (30). Thus, Kawasaki disease represents a pediatric-onset member of this disease spectrum. Management principles from these models—long-term inflammation control to reduce vascular risk—provide a framework for comprehensive Kawasaki disease care.

## 4 The clinical implications of a paradigm shift

### 4.1 Fundamental restructuring of patient management: from “acute-phase treatment + cardiac follow-up” to “lifelong vascular health management"

Redefining Kawasaki disease as a chronic condition necessitates a fundamental change in clinical management (Table 1). The traditional approach of acute-phase therapy followed by cardiac surveillance should evolve into a comprehensive, lifelong strategy for vascular health (21, 31). This shift begins by establishing a new risk stratification system. Current risk assessment, which relies primarily on coronary artery aneurysm status, is inadequate. A more accurate model should integrate multiple factors, including the severity of acute inflammation, specific immune responses, genetic predispositions, and traditional cardiovascular risk factors like blood pressure and lipid levels (32, 33). This integrated approach will improve long-term risk prediction (33). Subsequently, a structured lifelong monitoring plan is required.

**Table 1:** Implications of the chronic disease paradigm for risk assessment, monitoring, and intervention.

Domain	Traditional approach	Chronic disease model
Risk evaluation	Based mainly on coronary aneurysm	Multidimensional: inflammation severity, immune profile, genetics, classical cardiovascular risks
Monitoring target	Coronary arteries	Systemic vasculature (carotid, peripheral, stiffness)
Follow-up duration	Finite, pediatric-focused	Lifelong, transition to adult care
Biomarkers	Acute inflammation markers	Chronic inflammation, endothelial injury, metabolic risk
Intervention goal	Prevent aneurysm	Prevent atherosclerosis & vascular aging
Treatment philosophy	Episode-based	Preventive, continuous
Patient education	Disease recovery	Lifelong vascular protection

Monitoring should extend beyond the coronary arteries to assess systemic vascular health, including the carotid and peripheral arteries (34, 35). Both the frequency of monitoring and the methods used—such as ultrasound or biomarker testing—should be tailored to individual risk profiles (8).

For interventions, lifestyle modification forms the essential foundation, emphasizing diet, exercise, weight control, and smoking cessation (8). Pharmacological strategies also require reevaluation (8, 36). For high-risk individuals, long-term use of medications such as statins, low-dose aspirin, and potentially targeted anti-inflammatory agents (e.g., interleukin-1 inhibitors) may be beneficial (36). Future research should clarify the appropriate timing, duration, and patient selection for these treatments.

### 4.2 Transformation in clinician-patient communication and health education

Successfully implementing this new paradigm depends on transforming how clinicians communicate with patients and their families. It is essential to clearly explain that Kawasaki disease is a chronic vascular condition requiring ongoing management, not an illness that is cured after the acute phase (37). Education should focus on the link between the disease and long-term cardiovascular risk, helping patients understand why lifelong monitoring and healthy habits are necessary (8). Health education should therefore extend beyond disease-specific information to actively promote a vascular-healthy lifestyle (36). Empowering patients through knowledge and self-management skills is crucial for ensuring long-term adherence and improving outcomes (38).

## 5 Future research directions and challenges

### 5.1 Frontiers in basic research

Future research should focus on the key mechanisms that drive the transition from acute illness to chronic disease. A primary goal is to identify the “molecular switches” that initiate and sustain chronic pathology (20). Epigenetic changes, such as deoxyribonucleic acid methylation, may leave a lasting mark on immune and vascular cells (39). Researchers also need to study the specific memory immune cells formed during the acute phase, particularly those that persist in blood vessel walls and contribute to ongoing inflammation (40). Current animal models mainly mimic acute vasculitis; we urgently need new models that replicate chronic immune activation and early atherosclerosis for testing future treatments.

### 5.2 Priorities in clinical research

Clinical studies should translate the chronic disease concept into strategies that improve outcomes. A key focus is conducting trials to see if early treatment can prevent long-term vascular problems. For example, trials could test whether starting statins or targeted anti-inflammatory drugs early in high-risk patients reduces future risk (19, 41). Another priority is developing better tools to predict risk. This involves combining different types of data—such as blood tests, imaging, and genetic information—to create personalized risk scores and guide treatment decisions (42, 43).

### 5.3 Public health and policy considerations

Putting this chronic care model into practice requires changes in healthcare systems and policies. A major step is considering whether to include Kawasaki disease survivors in national heart disease screening programs (8). We should move beyond pediatric-only follow-up and build a lifelong care network (8). This network should connect pediatric, heart, rheumatology, and primary care doctors to ensure continuous care as patients become adults (37). Health economics studies are also needed to show that this lifelong approach is cost-effective, helping policymakers support these changes and ensure all patients receive proper long-term care (44, 45).

## 6 Summary

### 6.1 Integration of key arguments

In summary, viewing Kawasaki disease as a chronic condition reflects the natural progression of its underlying biology. This perspective acknowledges that the disease process continues long after acute symptoms resolve. The initial inflammatory response leads to lasting changes in immunity and blood vessel function. Over time, this contributes to faster vascular aging and higher heart disease risk. This chronic disease model explains long-term patient outcomes better than the traditional acute illness view.

### 6.2 Paradigm shift in clinical practice and patient care

This new understanding requires a fundamental change in how we care for patients. The goal shifts from treating a single acute episode to managing lifelong vascular health. Care should now focus on long-term prevention and monitoring, not just acute treatment. Each follow-up visit becomes an opportunity to assess and improve a patient's future cardiovascular health. This approach represents a move from reactive treatment to proactive health management.

### 6.3 A call to action: toward multidisciplinary lifelong health management

We urge researchers, clinicians, and health policymakers to embrace this new perspective. Moving forward requires collaboration across specialties including pediatrics, cardiology, and public health. Researchers should continue studying the long-term effects of Kawasaki disease, while clinicians develop practical lifelong care plans. Health systems should consider including Kawasaki disease survivors in heart disease prevention programs. Through coordinated efforts, we can improve lifelong health for all affected individuals.

## Data Availability

The original contributions presented in the study are included in the article/Supplementary Material, further inquiries can be directed to the corresponding author.
